# Evaluation of Plant Protein Hydrolysates as Natural Antioxidants in Fish Oil-In-Water Emulsions

**DOI:** 10.3390/antiox11081612

**Published:** 2022-08-19

**Authors:** Jeimmy Lizeth Ospina-Quiroga, Pedro J. García-Moreno, Antonio Guadix, Emilia M. Guadix, María del Carmen Almécija-Rodríguez, Raúl Pérez-Gálvez

**Affiliations:** Department of Chemical Engineering, University of Granada, 18071 Granada, Spain

**Keywords:** protein hydrolysates, antioxidant, DPPH scavenging activity, iron (II)-chelating activity, O/W emulsion, physical stability, lipid oxidation

## Abstract

In this work, we evaluated the physical and oxidative stabilities of 5% *w/w* fish oil-in-water emulsions stabilized with 1%wt Tween20 and containing 2 mg/mL of protein hydrolysates from olive seed (OSM–H), sunflower (SFSM–H), rapeseed (RSM–H) and lupin (LUM–H) meals. To this end, the plant-based substrates were hydrolyzed at a 20% degree of hydrolysis (DH) employing a mixture 1:1 of subtilisin: trypsin. The hydrolysates were characterized in terms of molecular weight profile and in vitro antioxidant activities (i.e., DPPH scavenging and ferrous ion chelation). After incorporation of the plant protein hydrolysates as water-soluble antioxidants in the emulsions, a 14-day storage study was conducted to evaluate both the physical (i.e., ζ-potential, droplet size and emulsion stability index) and oxidative (e.g., peroxide and anisidine value) stabilities. The highest in vitro DPPH scavenging and iron (II)-chelating activities were exhibited by SFSM–H (IC_50_ = 0.05 ± 0.01 mg/mL) and RSM–H (IC_50_ = 0.41 ± 0.06 mg/mL). All the emulsions were physically stable within the storage period, with ζ-potential values below −35 mV and an average mean diameter D[4,3] of 0.411 ± 0.010 μm. Although LUM–H did not prevent lipid oxidation in emulsions, OSM–H and SFSM–H exhibited a remarkable ability to retard the formation of primary and secondary lipid oxidation products during storage when compared with the control emulsion without antioxidants. Overall, our findings show that plant-based enzymatic hydrolysates are an interesting alternative to be employed as natural antioxidants to retard lipid oxidation in food emulsions.

## 1. Introduction

Polyunsaturated fatty acids (PUFA), and more specifically, those belonging to the omega-3 family such as eicosapentaenoic (C20:5n-3, EPA) and docosahexaenoic (C22:6n-3, DHA) acids, have attracted growing interest over the past decades as functional ingredients for food formulations. Several studies support the health benefits associated with a regular intake of omega-3 fatty acids, such as the prevention of cardiovascular disease [1], diabetes [2] and inflammatory diseases (e.g., asthma, rheumatoid arthritis, inflammatory bowel diseases and Alzheimer) [3,4], among others. EPA and DHA are semi-essential fatty acids since their natural conversion from α- linolenic acid is insufficient to meet human dietary requirements [5]. Therefore, both fatty acids should be supplied by diet. To this regard, the European Food Safety Authority (EFSA) recommends an adequate daily intake of 250–500 mg of EPA plus DHA for the maintenance of general cardiovascular health among healthy adults and children [6].

Among the different dietary sources for omega-3 PUFAs, fish oil is commonly used for food enrichment due to its unique composition and high digestibility. The incorporation of lipid ingredients, particularly PUFAs, into food matrices is hindered by their poor solubility and tendency to undergo oxidation. The oxidation of PUFAs results in undesirable fishy and rancid off-flavors, as well as reducing their potential health benefits [7]. To this regard, delivery systems such as oil-in-water (O/W) emulsions or capsules can provide a physical barrier between the lipids and oxygen or other pro-oxidant species. Although O/W emulsions are widely employed for liquid or meat-based foodstuffs fortified with fish oil [8,9,10,11], their oxidative deterioration during processing and storage (e.g., due to the increased specific surface area) still poses a major challenge. This drawback is commonly overcome by the use of antioxidant compounds, which are able to inhibit lipid oxidation by different mechanisms such as radical scavenging, metal ion chelation, reduction or singlet oxygen quenching, among others. Synthetic antioxidants (e.g., butylated hydroxyanisole, butylated hydroxytoluene, propyl gallate and tertbutyl hydroquinone) have been widely used in the food industry due to their strong antioxidant activity, availability and low cost. Nevertheless, their consumption presents several adverse side effects such as skin allergies, gastrointestinal disorders or even increased risk of cancer associated with long-term consumption. All these safety issues have promoted more stringent regulations restricting their use in favor of natural alternative compounds [12].

In general, antioxidant peptides are sequences containing 2–15 amino acids, which are inactive when encrypted within the pattern protein but display their biological activity when released by enzymatical hydrolysis, digestion or microbial fermentation. They share common features such as the presence of hydrophobic amino residues (i.e., Leu, Ile and Pro), aromatic amino acids such as Tyr or Trp or amino acids with nucleophilic sulfur-containing side chains (Cys and Met) [13]. These peptides find application as additives for food preservation, retarding the oxidation of food components, mainly lipids, during food processing and storage. Agro-industrial by-products are cheap and sustainable protein sources for antioxidant peptides. They present a variable protein content, ranging from 2% (e.g., potato peel) to more than 50% (e.g., soybean meal or pumpkin seed) in a dry weight basis [14]. Such materials have been extensively studied as substrates for releasing antioxidant peptides by enzymatic hydrolysis, employing commercial proteases such as alcalase, Flavourzyme, Protamex or papain. Enzymatic hydrolysis is a common method employed in the food industry to improve the nutritional and technological properties of proteins, as well as to obtain hydrolysates or peptides with biological activity. Compared with chemical hydrolysis, enzymes are specific catalysts which operate under moderate conditions of pH and temperature without generation of salts or other toxic by-products. These advantages make enzymatic hydrolysis the preferred treatment to produce bioactive peptides intended for food or feed applications. Indeed, antioxidant peptides have been identified in protein hydrolysates from a wide variety of plant substrates such as seeds [15,16], beans [17,18] or leaves [19,20], among others.

This work aims at investigating the potential use of plant protein hydrolysates (PPH) from sustainable sources as natural antioxidants for fish oil-in-water emulsions. To this end, four dried meals from olive seed, rapeseed, sunflower seed and lupin meals were hydrolyzed at 20% degree of hydrolysis (DH) by a mixture 1:1 of commercial proteases (i.e., subtilisin and trypsin). Most of these materials are generated as by-products from agriculture (i.e., lupin) or vegetal oil industry (i.e., olive, sunflower and rapeseed) and normally reduced to dried meals for animal feeding. The plant protein hydrolysates were characterized by their molecular weight distribution and in vitro antioxidant properties (i.e., radical scavenging and ferrous chelation activity) prior to their incorporation as natural antioxidants into 5% fish oil-in-water emulsions. The physical (i.e., droplet size distribution, zeta potential and creaming) and oxidative (peroxide and anisidine values) stability of the emulsions stabilized with Tween20 and containing the hydrolysates were monitored during a storage period of 14 days, with a special focus on the effect of PPH addition to retard oxidation of fish oil.

## 2. Materials and Methods

### 2.1. Plant Meals and Enzymes

This study employs four plant-based meals, obtained from local companies. Lupin (*Lupinus albus*) and olive (*Olea europaea*) seed meals were obtained from Dayelet (Barcelona, Spain) and Q’omer (Valencia, Spain), respectively. Sunflower (*Helianthus annuus*) seed and rapeseed (*Brassica napus*) meals were purchased from Bernabé Campal (Salamanca, Spain). All these plant substrates were analyzed for their protein content, reporting average values of 28.4, 20.9, 24.6 and 34.5%wt for lupin, olive seed, sunflower and rapeseed, respectively. According to the suppliers, the plant meals employed in this work presented a variable lipid content. While rapeseed and sunflower meal are by-products from oil extraction, so their fat content is below 4%wt, olive seed meal presented a reported lipid content between 8–14%wt, followed by lupin meal (7.2%wt). The plant protein hydrolysates were produced employing two commercial endoproteases, Alcalase 2.4 L (subtilisin EC 3.4.21.62) and PTN 6.0S (trypsin 3.4.21.4), both purchased from Novozymes (Bagsvaerd, Denmark). All the analytical-grade chemicals employed for analysis were purchased from Sigma-Aldrich (Merk, New York, NY, USA).

### 2.2. Enzymatic Hydrolysis

The enzymatic treatments were carried out in a jacketed glass reactor at a laboratory scale. To this end, samples were homogenized in distilled water at a ratio 2.5% protein *w/v*. This suspension was transferred to a 250 mL jacketed reactor heated at 50 °C under magnetic stirring. After that, pH 8.0 was adjusted and maintained throughout the reaction, employing 0.5 M sodium hydroxide by means of a pH-stat titrine (718 STAT Titrino, Metrohm, Switzerland). An enzymatic mixture containing Alcalase 2.4 L and PTN (1:1, *w/w*) was employed as catalyst, employing an enzyme-to-substrate ratio of 5% (*w/w*). All the hydrolysis reactions were performed in duplicate to confirm the reproducibility of the hydrolysis curves. The enzymatic reactions were allowed until attaining a degree of hydrolysis of 20%. At this point, the hydrolysis was stopped by heating the reaction mixture at 90 °C for 10 min, to inactivate enzymes. Finally, the final hydrolysates were vacuum-filtered and then freeze-dried in a LyoMicron lyophilizer (Coolvacuum Technologies, Barcelone, Spain). The dried powders obtained from two replicated hydrolysis were mixed and stored at −20 °C prior to analysis.

The degree of hydrolysis (DH) can be related to the amount of 0.5 N NaOH added to the reaction mixture to keep pH constant during the enzymatic treatment, according to the pH-Stat method [21] (pp. 122–123) as shown in Equation (1):

(1) $$DH=\frac{V_{b}\cdot N_{b}}{\alpha \cdot m_{P}\cdot h_{tot}}$$

where *V_b_* (mL) and *N_b_* (eq/L) are the volume and normality of the base employed for the titration, respectively. The term m_p_ stands for the mass of the protein present in the reaction mixture (g), *h_tot_* represents the number of peptide bonds per mass of protein and was assumed as 8.6 milliequivalents of peptide bonds per gram of protein [21] (pp. 146–147). The average degree of dissociation of the α-amino groups (*α*) at pH 8.0 and 50 °C was estimated as 88.5%, as reported in the literature [21] (p. 142).

### 2.3. Plant Meal Solubilization and Protein Recovery

After enzyme inactivation, all hydrolysates were vacuum-filtered through 8 mm cellulose paper, and the solids retained were dried in an oven at 105 °C for 2 h. The percentage of solubilization of the plant meals in water after the enzymatic treatment was calculated by Equation (2):

(2) $$\%\;Solubilization=\left(1-\frac{m_{R}}{m_{0}}\right)\cdot 100$$

where *m_R_* (g) represents the mass of dried solids retained on the filter paper, and *m*_0_ (g) is the mass of plant meal dissolved in distilled water at the start of the reaction.

Similarly, protein recovery, defined as the ratio of protein present in the hydrolysate to that in the original substrate, was estimated by Equation (3). This variable is an index of the protein solubilization attained after the enzymatic treatment.

(3) $$\%\;Protein\;recovery=\left(\frac{m_{H}\cdot c_{H}}{m_{0}\cdot c_{0}}\right)\cdot 100$$

where *m*_0_ and *m_H_* stand for the mass of plant meal and freeze-dried hydrolysate, which present protein weight fractions (g of protein/g of dried sample) denoted by *c*_0_ and *c_H_*, respectively.

### 2.4. Characterization of the Hydrolysates

#### 2.4.1. Proximate Composition of the Hydrolysates

The freeze-dried hydrolysates were analyzed for their protein, lipid and moisture content. A Flash 2000 CHNS/O elemental analyzer (Thermo Scientific, Waltham, MA, USA) was employed to determine the protein content of meals and hydrolysates. Here, the samples are subjected to complete combustion, and a thermal conductivity detector identified the electrical signal of combustion products (CO_2_, H_2_O, N_2_ and SO_2_), which is proportional to each elemental concentration (C, H, N and S). The nitrogen-to-protein content factor was assumed to be 5.3, following the work of Rhee K [4].

The lipid content of the PPH was determined after four sequential extractions with a hexane and 2-propanol mixture (1: *v/v*). Briefly, a certain amount of hydrolysate (between 0.2 and 1.0 g) was mixed with 5 mL distilled water, 20 mL of solvent mixture and then vortexed for 5 min. After that, it was centrifugated at 1200× *g* for 5 min. The supernate phase was taken to evaporate solvent, and the oil content was quantified by dividing oil mass per hydrolysate mass used.

The moisture content of the PPH was determined by means of an infrared moisture analyzer (AD-4714A, A&D Company, Oxford, UK).

#### 2.4.2. Molecular Weight Distribution of the Hydrolysates

The molecular weight distribution of the hydrolysates was obtained by size exclusion chromatography (SEC). To this end, the hydrolysates were dissolved in distilled water at 10 mg/mL, and 500 μL of each sample was injected into a Superdex Peptide 10/300 GL column (GE Health-care, Uppsala, Sweden) for elution using distilled water as a mobile phase at 0.5 mL/min. The absorbance of the eluted sample was measured at 280 nm. The calibration curve was prepared using different standards with broad weight size distributions (L-Tyrosine (217.7 Da), Vitamin B_12_ (1355.4 Da), Aprotinin (6512 Da), Cytochrome C (12,384 Da) and Ribonuclease A (13,700 Da).

#### 2.4.3. Antioxidant In Vitro Activity of Hydrolysates

Two antioxidant properties were investigated in the plant protein hydrolysates, namely the ability to sequestrate 2,2-diphenyl-1-picrylhydrazyl (DPPH) radicals and the ferrous ion chelating activity.

The DPPH scavenging activity was determined by the method of Picot et al. [22] with slight modifications. Briefly, each hydrolysate aqueous solution was mixed and shaken with the same volume of 0.1 mM DPPH methanolic dissolution. The mixtures were kept for 30 min at room temperature in the dark before measuring absorbance at 515 nm. A series of control samples were prepared by mixing 1 volume of hydrolysate solution with 1 volume of methanol. The blank solution, which displays the minimum radical scavenging, was a mixture 1:1 of DPPH and distilled water. The radical scavenging inhibition was calculated as follows:

(4) $$DPPH\;inhibition,\;\%=\left(1-\frac{A_{sample}-A_{control}}{A_{blank}}\right)\cdot 100$$

where *A_sample_*, *A_control_* and *A_blank_* denote the absorbances for the sample, the control samples (without addition of DPPH) and the blank solution (DPPH plus distilled water), respectively.

This procedure was repeated for a serial dilution of hydrolysates (0.1–20 mg protein/mL), allowing the calculation of the half-maximal inhibitory concentration (IC_50_, mg/mL) for each hydrolysate.

Ferrous ion chelating capacity is related to the ability of the plant protein hydrolysates to chelate free metal cations, which catalyze lipid oxidation reactions. This property was determined by the method reported by Decker and Welch [23]. Each aqueous hydrolysate dissolution was mixed with distilled water (1:3.7, *v/v*) and 0.1 mL of 2 mM ferrous chloride aqueous dissolution. After 3 min, 0.2 mL of 2 mM Ferrozine aqueous dissolution was added to all samples except those used as control, and the blank was analyzed using distilled water instead of hydrolysate dissolution. After incubation for 10 min, the absorbance was read at 562 nm. The iron (II)-chelating activity was then calculated according to Equation (5):

(5) $$Fe\left(II\right)\;chelating\;activity,\;\%=\left(1-\frac{A_{sample}-A_{control}}{A_{blank}}\right)\cdot 100$$

where *A_sample_*, *A_control_* and *A_blank_* denote the absorbances for the sample, the control sample without addition of Ferrozine and the blank solution where the hydrolysate was replaced by distilled water, respectively.

For comparison purposes, the in vitro chelation of the hydrolysates was expressed as half-maximal concentration (IC_50_, mg/L).

### 2.5. Preparation of Emulsions

Six emulsions were prepared to contain 5.0% (*w/w*) of refined fish oil (BASF Personal Care and Nutrition GmbH, Illertissen, Germany) and stabilized with 1.0% (*w/w*) Tween 20^TM^. Four emulsions were produced by incorporating the plant protein hydrolysates as antioxidants into the aqueous phase at a concentration of 2 mg/mL. They were coded as LUM–H (lupin meal hydrolysate), OSM–H (olive seed meal hydrolysate), SFSM–H (sunflower seed meal hydrolysate) and RSM–H (rapeseed meal hydrolysate). Additionally, a positive control emulsion was prepared with whey protein hydrolysate at DH 10% (WPC–H) instead of plant protein hydrolysate. We confirmed by previous studies [24,25] the ability of whey protein hydrolysates to retard lipid oxidation, supporting their use as natural antioxidants in fish oil-in-water emulsions. Finally, a negative control emulsion was produced without the addition of any antioxidant.

Firstly, the aqueous phase containing the protein hydrolysate and the emulsifier Tween 20^TM^ was brought to pH 8.0 and left stirring overnight at 4 °C to allow solubilization and rehydration of the protein. Pre-emulsions were prepared by dispersing the fish oil in the aqueous phase by means of an Ultra Turrax mixer (IKEA Werke GmbH & Co., Staufen, Germany) at 15,000 rpm for 2 min. Then, homogenization was conducted in a high-pressure laboratory homogenizer (Panda Plus 2000, GEA Niro Soavi., Lübeck, Germany) at 450/75 bar, running 3 passes. To accelerate lipid oxidation, 100 µM of FeSO_4_ 7 H_2_O was added to the emulsions. Additionally, 0.05% (*w/w*) of sodium azide was incorporated into the emulsions to avoid microbial growth. The emulsion samples were stored in amber glass jars at 25 °C in the dark for 14 days. To evaluate lipid oxidation in the emulsions (i.e., peroxide and anisidine value), sampling was carried out on days 0, 1, 3, 7 and 14. As for the physical stability, samples were drawn on days 0 and 14 for measuring droplet size distribution, creaming and Turbiscan Stability Index (TSI), while zeta potential was measured on day 1.

### 2.6. Physical Stability of the Emulsions

The droplet surface charge was determined by measuring the zeta potential (ζ, mV). To this end, the emulsions were diluted 1:500 *v/v* in distilled water, and then pH was adjusted to 8. The zeta potential was measured at room temperature by means of a Zetasizer Nano ZS (Malvern Instruments Ltd., Worcestershire, UK) with a DTS-1060C cell. The zeta was set in the range between −100 and 50 mV, conducting three replicated measurements.

The oil droplet size distribution of the emulsions was obtained by means of laser diffraction equipment, employing a Mastersizer 3000 (Malvern Instruments Ltd., Worcestershire, UK), where samples were scattered in recirculating water at 3000 rpm until reaching an obscuration in the range of 12–15%, and the refractive indices employed for fish oil and water were 1.481 and 1.330, respectively. The results are reported as surface area D_3.2_ and volume mean D_4,3_ diameter. The physical stability of the emulsions was additionally evaluated by multiple light scattering in a Turbiscan^TM^ LAB analyzer (Formulaction., Toulouse, France). To this end, 25 mL of each emulsion were reserved in a glass cell to perform measurements of the TSI. Finally, the emulsion destabilization during storage was further evaluated by placing 10 mL of each emulsion in graduated glass tubes and calculating the creaming index as the percentage of phase separation [26].

### 2.7. Oxidative Stability of the Emulsions

#### 2.7.1. Determination of the Peroxide Value

Firstly, the lipid fraction from emulsions was extracted according to Padial-Domínguez et al. [24] with minor changes. As extraction agents, 20 mL of 1:1 *v/v* of hexane and 2-propanol were employed. Circa 0.5 g of emulsion (containing around 20 mg of fish oil) was homogenized with 5 mL of distilled water, vortexed for 5 min and then centrifugated at 1200× *g* for 4 min. Two extractions were made from each emulsion.

The Peroxide Value (PV) was determined according to the standard method described by Shantha N. and Decker E. [27] with minor modifications. In short, oil extracted from emulsions was mixed with 5 mL of 2-propanol, 50 µL of ammonium thiocyanate and 50 µL of iron (II) dissolution. The samples were vortexed and incubated for 5 min at 25 °C. After, absorbance was measured at 485 nm in a Genesys^TM^ 30 visible spectrophotometer (Thermo Fisher Scientific, Whalham, MA, USA). Four replicates were performed, and results are presented in milliequivalents of peroxide per kilogram of oil.

#### 2.7.2. Anisidine Value Assay

The p-anisidine method was carried out according to the ISO 6885:2006 method [28] with slight modifications. In brief, the lipid extract was mixed with 10 mL of hexane, vortexed and distributed into two Pyrex^TM^ tubes with screw caps at equal volumes. In the first tube, 1 mL of anisidine dissolution (2.5 mg/mL) in glacial acetic acid was added, while 1 mL of hexane was incorporated in the other one as the control sample. All of them were covered and left in the dark for 10 min before measuring absorbance at 350 nm in a 10 mm cell.

### 2.8. Statistical Analysis

The software RStudio 2022.02.1 (RStudio Team, Boston, MA, USA) was employed to conduct one-way analysis of variance in the data. The significant differences among samples and treatments were computed according to Tukey’s multiple comparison test. Differences between means were considered significant at a level of confidence of 95% (i.e., *p*-value below 0.05).

## 3. Results and Discussion

### 3.1. Characterization of the Hydrolysates

The proximate composition of all the plant protein hydrolysates, reported in terms of protein, lipid and moisture content, is shown in Table 1. The plant meals employed as substrate for the hydrolysis presented an average protein content between 20.9%wt (olive seed meal) and 34.5%wt (rapeseed meal), which make them suitable as substrate to produce protein hydrolysates. All these substrates were chosen based on their protein content and the previous literature supporting their functional properties. For instance, white lupin is a legume with a reported protein content between 28 and 44%wt, which could promote health benefits due to its bioactive compounds, such as anti-inflammatory, antidiabetic, antioxidant and antihypertensive peptides [29,30]. Likewise, oilseed by-products, with a raw protein content of over 20%wt, have been gaining importance as a source of phytosterols, tocopherols and phenolic compounds, as well as peptides with biological activity [31]. In addition to protein, plant-derived meals are sources for a variety of compounds such as alkaloids, polyphenols, vitamins and minerals, as well as lipids, starch and fiber. The latter is usually present as the major component in plant meals [32,33], being responsible for their limited water solubility ([34,35], p. 364). The enzymatic treatment led to improved solubilization of the plant meals, with observed values ranging from 57.9% (lupin) to 75.5% (rapeseed) (Table 1). The limited solubility of plant meals could restrain their use as functional ingredients in food and nutraceutical preparations [31,36]. To this regard, Nissen, A. ([21], pp. 102–103) and Mokni, G. et al. [37] concluded that the enzymatic hydrolysis with alcalase significantly improved protein solubilization. The cleavage of peptide bonds leads to higher exposure of polar groups, and therefore higher solubility of the hydrolysate compared with the native protein. Similar to alcalase, trypsin cleaves preferably the C-terminus of Arg or Lys residues, releasing positive-charged peptides with improved solubility [38,39]. This is reflected in average values of protein recovery for the hydrolysates, as well as their protein content compared with the plant flours, which suggests that enzymatic reaction was effective to solubilize protein in the reaction medium. Sunflower seed meal hydrolysate presented the highest protein recovery in the experimental series (82.2%), similar to rapeseed meal hydrolysate. These results are in agreement with Vioque et al. [40], which mention that the use of oilseed proteins is frequently limited by their low solubility but could be offset by enzymatic hydrolysis.

Moreover, these results are in agreement with other protein-content sources of different origins. For example, Rivero-Pino et al. [41] studied proteins from *Tenebrio Molitor* as a source of bioactive peptides. They found that enzymatic hydrolysis improved protein solubility in contrast with unhydrolyzed protein. Over all treatments, alcalase–flavourzyme led to highest protein recovery, obtaining 46% at DH 20%.

**Figure 1:**
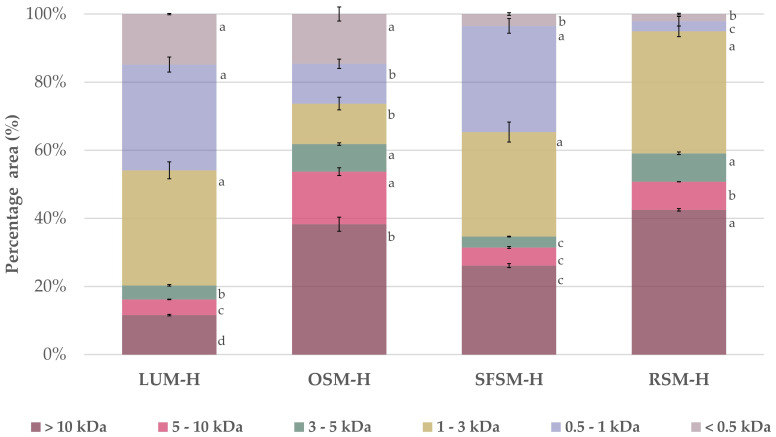
Relative molecular weight distribution for the four plant protein hydrolysates. LUM–H: lupin meal hydrolysate; OSM–H: olive seed meal hydrolysate; SFSM–H: sunflower seed meal hydrolysate; RSM–H: rape-seed meal hydrolysate. All the data are expressed as mean ± standard deviation of triplicate measurements. Different superscript letters indicate significant differences (*p* < 0.05) among plant protein hydrolysates.

The molecular weight (MW) profile distribution of the four hydrolysates (Figure 1) confirms that the enzymatic treatment with alcalase and PTN was effective to hydrolysate the plant proteins, releasing a distribution of peptides. Alcalase is a broad-spectrum endoprotease, which is widely reported in the literature as a catalyst for the production of food protein hydrolysates [42]. Overall, we found a high presence of low MW peptides below 3 kDa in LUM–H and SFSM–H with a relative proportion of 79.7 and 65.3%, respectively. Additionally, RSM–H and OSM–H exhibited a major proportion of peptides above 5 kDa with 50.7 and 53.7%, respectively. As stated by Ying X., et al. [43], most of the bioactive peptides identified so far have 2–20 amino acid residues, with an average molecular weight under 6 kDa. More specifically, it has been observed that bioactive peptides displaying antioxidant capacity have short chains with a low molecular weight within 0.4 and 2 kDa [43].

### 3.2. Antioxidant In Vitro Activity of Hydrolysates

The antioxidant characteristics of peptides have been related to their ability to scavenge free radicals, chelate metal ions, or act as physical shieldings [44,45]. In the first case, peptides can act as radical inhibitors by donating electrons while holding their stability through the resonance of their structure. Meanwhile, peptides with carboxyl and amino groups on their side chains have a chelating function of metal ions as they can dissociate and be proton donors, inhibiting the production of free radicals through stabilizing metallic pro-oxidants. In addition, they can also act as a physical barrier or membrane to inhibit lipid peroxidation due to their surfactant properties, reducing direct contact between lipids and radicals and other oxidizing species [45].

**Figure 2:**
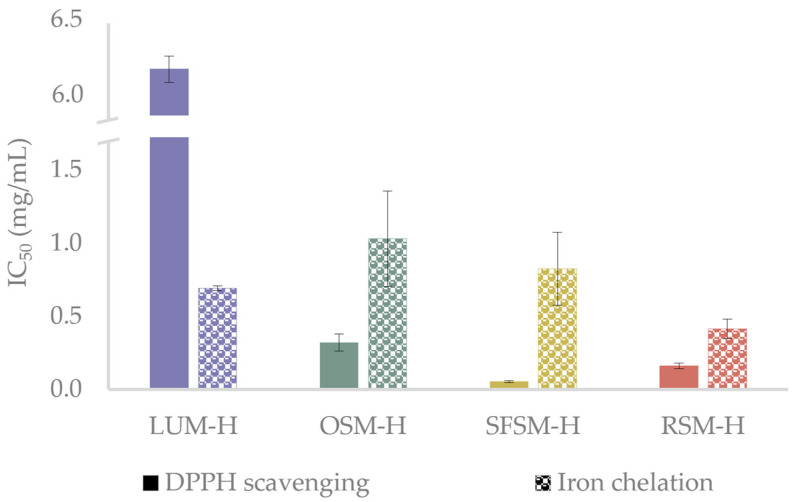
DPPH scavenging and iron (II)-chelating activity of the hydrolysates, reported as IC50 value (mg/mL). LUM–H: lupin meal hydrolysate; OSM–H: olive seed meal hydrolysate; SFSM–H: sunflower seed meal hydrolysate; RSM–H: rape-seed meal hydrolysate.

As radical quenching is the primary mechanism employed by antioxidant compounds to inhibit oxidation processes [46], and metals are initiators of undesired oxidative reactions in food products [47], DPPH radical scavenging and iron (II) chelation assays were employed to test the antioxidant in vitro capacity of the hydrolysates (Figure 2). The antioxidant in vitro assays reveals an outstanding capacity of the hydrolysates to scavenge DPPH radicals, with IC_50_ values ranging from 0.05 to 6.19 mg/mL. SFSM–H and RSM–H exhibit a significantly higher radical scavenging activity over OSM–H and LUM–H, with this last one showing the lowest activity among all samples (*p* < 0.05). Ying et al. [43] describe Tyr, Lys, Phe, Arg and Met as amino acid residues that could confer strong antioxidant properties. Wang et al. [48] mention that Val, Cys, Phe and Trp have been considered to have a good antioxidant capacity to quench free radicals or reduce metal ions, in agreement with Manzoor et al. [44] and López-García et al. [45], who reported amino acids such as Tyr, His, Trp and Phe as potent radical inhibitors. Bougatef et al. [49] found in their study with *Mustelus mustelus* muscle protein hydrolysates that the best DPPH radical-scavenging activity was exerted by peptides with MW below 3.5 kDa, with an IC_50_ close to 0.25 mg/mL. The aminogram results of this fraction revealed an important presence of His, Met, Tyr, Leu, Ile, Gly and Arg. Based on these investigations and the amino acid profiles reported for LUM, OSM, SFSM and RSM, we can suggest that prominent DPPH inhibition activity of oilseed by-products hydrolysates could be conferred due to the presence of some specific amino acids in the raw material. For example, OSM–H may have an important amount of Tyr (25%), Glu (7.5%), Met (6.8%), Arg (5.1%) and Pro (5.1%) [50]; SFSM–H of Arg (8.5%), Leu (7.0%), Gly (6.3%) Val (5.8%), Ile (4.9%) and Pro (4.3%) [51]; and RSM–H of Gly (10.3%), Leu (8.2%) and Arg (6.2%) [52]. Regarding LUM–H results, it is possible to find some presence of Arg (10.7%), Phe + Tyr (8.7%) and Leu (6.8%) [53,54]. Nevertheless, although meals in this work have been reported with amino acids that could confer antioxidant activity, it is important to remark that protein meal solubilization in hydrolysis could change the amino acid profile of all hydrolysates. Additionally, other factors strongly influence the peptide antioxidant activity and should be taken into account, such as the position of some amino acids within the peptidic sequence, peptide chain length and the ability of some hydrophobic amino acids (e.g., Pro, Val, Leu and Tyr) to favor the interaction of peptides with hydrophobic radical species formed at the lipid phase [44,55]. The highest DPPH inhibition performed by SFSM–H, in comparison with the other hydrolysates, could be related to a major presence of peptides below 3 kDa. The low IC_50_ values for DPPH inhibition reached in our hydrolysates agree with those of He et al. [56], who evaluated the action of different enzymes over antioxidant activities of rapeseed protein isolate. They found that alcalase was one of the most efficient enzymes to produce antioxidant peptides, with DPPH values between 0.4–0.8 mg/mL. However, it should be noted that peptide hydrolysates are not purified, and therefore they contain other compounds that could confer antioxidant activity. For example, Wang et al. [48] studied the fermentation of RSM, finding that DPPH radical scavenging results are not only due to the presence of low MW peptides but the liberation of phenolic compounds that can take place by proteases action. Likewise, González-Hidalgo et al. [57] studied the antioxidant ability of olive by-products, finding that olive seeds showed an interesting amount of phenolic compounds (hydroxytyrosol, tyrosol and oleuropein) which exert high antioxidant activity.

The Fe^2+^ chelating assay showed IC_50_ values ranging between 0.41 and 0.97 mg/mL. RSM–H displays a significantly higher capacity to chelate Fe^2+^ over OSM–H, SFSM–H and LUM–H. These IC_50_ values are similar to those reported for the peptide fractions from *Gadus morhua* obtained by Sabeena Farvin et al. [58], which exhibited values ranging from 0.15 to 0.75 mg/mL (crude hydrolysate). The further investigations on *Gadus morhua* hydrolysates [55] suggested that presence of Glu, Gly, Lys, Ala, Arg, His, Tyr, Phe and Pro amino acids may contribute to the iron chelation displayed by the hydrolysates. Other works have reported similar trends, such as those carried out by Zhang et al. [59], which evaluated the antioxidant ability of hydrolysates from chickpea protein and Carrasco-Castilla et al. [60] from bean protein hydrolysates. These studies mention that Glu and Arg, as well as Asp+Asn, Glu+Gln, His and Cys, display an important role when it comes to the chelation of metal ions. Moreover, low MW peptides below 3 kDa could be responsible for the good general performance of SFSM–H and RSM–H among the samples, in agreement with Sabeena Farvin et al. [58], which evaluated the antioxidant activity of cod protein hydrolysates both in vitro and over oil-in-water emulsions with 5% of fish oil. They found that peptides below 3 kDa displayed the lowest IC_50_ (≈0.15 mg/mL). Based on these investigations, and the reported amino acid composition of our hydrolysates, Glu, Gly and Asp seem to act as important chelating agents in RSM–H. However, Glu and Asp could also be representative inside LUM and SFSM, suggesting that Gly may be responsible for the higher chelating activity of RSM–H, in contrast with the other samples. Nevertheless, OSM–H also exerts important results in this test, which could be due to the high presence of Tyr.

### 3.3. Physical Stability of Emulsions

The physical stability of the emulsions was studied during 14 days of storage, as shown in the Table 2. All the emulsions produced exhibited high physical stability over storage time. The ζ-potential was measured as an indicator of the charge of the dispersed oil droplets in the emulsion, which is related to its physical stability [51]. High absolute values above 30 mV have been linked with sufficient electrostatic repulsion between droplets that might lead to physically stable emulsions [52]. All the emulsions presented a negative ζ-potential below—40 mV except for the control emulsion NC and WPC–H, which present no significant statistical differences among them. Even if Tween20 is a non-ionic surfactant, Yesiltas et al. [61] suggest that negative ζ-potential values could be due to traces of free fatty acids or other anions present in the buffer. To this regard, we should note the presence of OH^-^ anions added as titration agent (i.e., NaOH) to maintain pH at 8.0 during the hydrolysis. Moreover, the high negative values of zeta potential observed in some the emulsions containing the plant hydrolysates could be related to other natural compounds present in the plant source, such as phenols or gums [62].

Regarding the droplet size distribution, all the emulsions showed a monomodal distribution centered around 0.4 μm with no significant differences from day 0 to day 14 (Figure S1), indicating high physical stability. The statistical diameters D[3,2] and D[4,3] are listed for day 14 on the Table 2. The surface area mean diameter D[3,2] at day 14 ranged from 0.312 to 0.329 μm among the plant protein hydrolysates, while the volume mean diameter D[4,3] varied from 0.393 to 0.425 μm. According to Tukey’s test, there were not statistical differences between the statistical diameters evaluated at day 0 and 14, presenting slight deviations attributed to the experimental procedure (e.g., emulsion preparation and homogenization). The D[4,3] diameters of our emulsions were higher than those reported by Betül Yesiltas et al. [61], who reported a range between of volume mean diameters between 0.188–0.229 μm. These differences could be explained by the different equipment employed for the homogenization step in the two studies (two-valve homogenizator versus microfluidizer).

**Figure 3:**
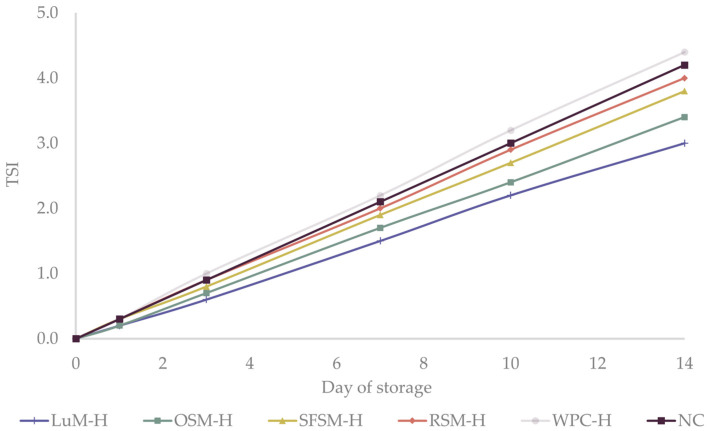
Turbiscan stability index (TSI) of the six emulsions during storage. LUM–H: lupin meal hydrolysate; OSM–H: olive seed meal hydrolysate; SFSM–H: sunflower seed meal hydrolysate; RSM–H: rapeseed meal hydrolysate; WPC–H: whey protein concentrate hydrolysate; NC: negative control (without hydrolysate).

Turbiscan stability index (TSI) is another useful parameter to study the physical destabilization of emulsions over time. TSI values below 3 are an indicator of high physical stability [61]. During the first 7 days of the study, TSI of all emulsions (Figure 3) slightly raised between 1.5–2.2, reaching values between 3.0–4.2 on the last day. These findings are in agreement with creaming experiments, which did not present a visual destabilization through the storage time (data not shown). Additionally, our results are similar to those obtained by Betül Yesiltas et al. [61] for 5%wt fish oil-in-water emulsions stabilized with 1 %wt Tween20 containing synthetic antioxidant peptides from different sources. Overall, we concluded that the emulsions were physically stable over the storage period, presenting similar droplet sizes. Physically stable emulsions were required in order to explain the differences in oxidative stability among emulsions based on the addition of the plant protein hydrolysates.

### 3.4. Oxidative Stability of Emulsions

#### 3.4.1. Peroxide Value

Lipid oxidation critically determines the chemical stability of fish oil-in-water emulsions. Oxidation of fish oil generates a wide variety of compounds, hydroperoxides being the primary oxidation products. As hydroperoxides are highly unstable compounds, they could be decomposed by the action of heat and traces of metals to secondary or final oxidation compounds, which are complex mixtures of volatile, non-volatile and polymeric, among other compounds [61]. Proteins and peptides incorporated into emulsions could inhibit lipid oxidation in different ways: (i) peptides not adsorbed at the interface can bind metal ions and scavenge free radicals in the aqueous phase, whereas (ii) adsorbed peptides could repel cationic metal ions from the interface or bind radicals formed in the proximity of the interfacial layer [43]. A similar droplet size distribution observed for all the emulsions (Table 2 and Figure S1) indicates that the hydrolysates did not show a significant emulsifying activity, denoting that the peptides added to the emulsions were mainly located in the aqueous phase. In this work, the peroxide value and anisidine index were employed to determine primary and secondary (unsaturated aldehydes) oxidation products in the emulsions during 14 days of storage.

**Figure 4:**
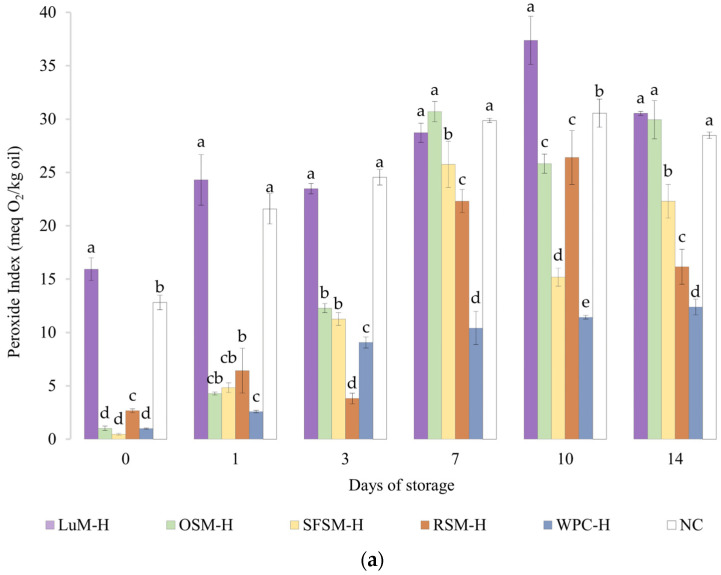

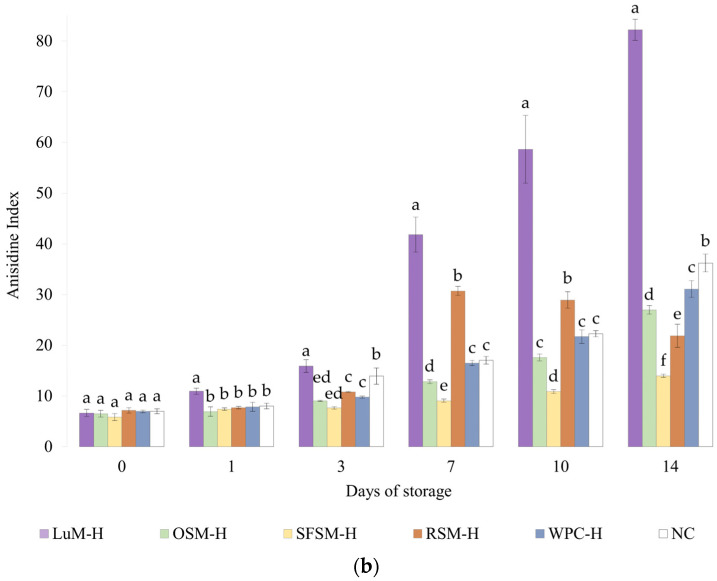
(**a**) Peroxide and (**b**) anisidine value for emulsions prepared with plant-based hydrolysates as antioxidant compounds. LUM–H: lupin meal hydrolysate; OSM–H: olive seed meal hydrolysate; SFSM–H: sunflower seed meal hydrolysate; RSM–H: rapeseed meal hydrolysate; WPC–H: whey protein concentrate hydrolysate; NC: negative control (without hydrolysate). All the data are expressed as mean ± standard deviation of triplicate measurements. Different superscript letters indicate significant differences (*p* < 0.05) among plant protein hydrolysates.

The peroxide value (PV) of emulsions is shown in Figure 4a. The emulsion without antioxidants (NC) had a PV of 12.8 meq O_2_/kg oil after production, reaching a maximum (36 meq O_2_/kg oil) on day 7. From this point on, the average values of PV tend to decrease, which implies a lower rate of formation than decomposition of peroxides. These results are comparable with negative control emulsion by Yesiltas et al. [61], which was prepared similarly to ours. In the latter, the first and last (eighth) day of stability had a PV close to 15 and 57 meq O_2_/kg oil. Moreover, the trends shown by our emulsions were similar to those reported by Cheng et al. [63] in emulsions with 10%wt of soybean oil, 1.13%wt of Tween20 and no antioxidant incorporated.

In contrast, the emulsion containing WPC–H, which was previously reported to exhibit antioxidant activity, had a PV of 1.0 meq O_2_/kg oil after production. This emulsion showed a 1-day lag phase, and then PV linearly increased to reach its maximum value of 12.4 meq O_2_/kg oil on day 14. It should be noted that the emulsion containing WPC–H showed significantly lower PV values than the control emulsion without antioxidants. Our results are higher but similar in trend to Padial-Domínguez et al. [24], which produced emulsions using 2%wt of WPC–H as emulsifier instead of Tween20.

Regarding the emulsions containing the plant-based hydrolysates, the emulsion with LUM–H exhibited the highest PV in the experimental series, remaining above NC emulsion all over the storage period. The lower oxidative stability of the emulsion with LUM–H could be explained due to the lowest in vitro antioxidant activity of LUM–H (Figure 2) together with a very negative zeta potential of this emulsion (Table 2). The surface net charge observed on day 1 and mean diameters computed for the droplet distributions could favor the attraction of Fe^2+^ to the interface catalyzing lipid oxidation [64]. Emulsions containing OSM–H and SFSM–H showed similar PV values during storage, reaching their maximum on day 7 (30.7 and 25.8 meq O_2_/kg oil, respectively), with considerably lower PV values when compared with the emulsion without antioxidants. Interestingly, the PV of the emulsion containing RSM–H displayed low PV and a lag phase until day 3. Then, the PV rapidly increased and tended to reduce between days 10 and 14. Overall, although emulsions containing hydrolysates from oilseeds by-products were not better in contrast with the emulsion containing WPC–H, they were able to delay the primary oxidation of fish oil by staying below the emulsion without antioxidants in most of the storage days. To the best of our knowledge, there are no works to date that evaluated the oxidative oil-in-water stability of hydrolysates from LUM, OSM, SFSM and RSM, and very few are found from other sources. This is the case of Cheng et al. [63], who studied the oxidative stability of soybean (10%wt) oil-in-water emulsions over 14 days using potato hydrolysates treated with alcalase as natural antioxidants and Tween20 (1.13%wt) as emulsifier. The authors tested different levels of plant protein hydrolysates, reporting minimal oxidation (PV around 20 meq O_2_/kg oil at day 14) for the emulsion containing 20 mg/mL of hydrolysate. A similar change in PV was observed for SFSM–H (PV ranging from 0.5 to 22.3 meq O_2_/kg oil) and RSM–H (PV ranging from 2.7 to 16.2 meq O_2_/kg oil). In contrast, it is noticeable that in our study we used 2 mg/mL of hydrolysate as antioxidant ingredient.

#### 3.4.2. Anisidine Value

The Figure 4b shows the p-anisidine value (AV) of emulsions containing plant-based hydrolysates and the control emulsions. Like the PV results, LUM–H shows the highest AV values, remaining above the NC emulsion during the storage period. These results denote the poor oxidative stability of LUM–H emulsion, which could be explained by its low in vitro antioxidant activity and more negative zeta potential, which favors metal-catalyzed oxidation. Moreover, the lipid content of the lupin hydrolysate (5.9 %wt, Table 1) could also contribute to initiate lipid oxidation [24]. Regarding the RSM–H emulsion, the AV value surpassed the control emulsion WPC–H from the day 7 on, remaining stable until the final storage day. This behavior was unexpected due to the good in vitro DPPH scavenging activity and Fe^2+^ chelation capacity observed. However, in vitro tests do not represent all complex lipid oxidation mechanisms that take place in the emulsion [65], which could explain the differences between in vitro and emulsion results. Ka et al. [66] evaluated the anti and pro-oxidant properties in vitro and in oil-in-water emulsions of some amino acids, and one of their findings was that some residues (e.g., Cys) exert high antioxidant in vitro capacity but not in oil-in-water emulsions, suggesting that high in vitro antioxidant capacity will not necessarily display similar antioxidant capacities in a real food matrix. By contrast, OSM–H and SFSM–H were able to retard the formation of unsaturated aldehydes in emulsions to greater extent when compared with the control emulsion (NC) or the emulsion containing WPC–H. OSM–H considerably increases the AV after 7 days of storage but without surpassing NC. Shi et al. [67] mention that Tyr, Trp, Met and Cys amino acids confer excellent antioxidant capacities to hazelnut protein peptides, acting as important hydrogen donors for free radicals. Among these amino acid residues, they found that Tyr-containing peptides exert a significant antioxidant activity. In addition, López-García et al. [45] also reported Val as a good electron donor, which could be present in an important amount in OSM–H. Nevertheless, they also mentioned that certain amino acids such as His, Tyr, Trp, Met, Cys and Pro could also avoid lipid peroxidation, which may explain the OSM–H behavior on lipid oxidation tests, as it could be possible to find a big proportion of Tyr and Val in the raw material, as well as some presence of Met. The latter indicates that these hydrolysates possess the ability to reduce the decomposition of peroxides to secondary oxidation products. This was especially the case of SFSM–H since the AV remains quasi-constant, with a slight increment during storage. It is worth noting that SFSM–H was more capable of retarding the secondary oxidation in contrast with WPC–H. The latter could be due to the presence of peptides with antioxidant character but also may be to the presence of other antioxidant compounds (e.g., flavonoids) [68]. In addition, a higher DH in SFSM–H (20%) could lead to the formation of lower peptide chain lengths compared with the DH of WPC–H (10%). This good oxidative stability is in agreement with in vitro results, where SFSM–H displays a remarkable scavenging activity.

## 4. Conclusions

All plant-based hydrolysates employed in this study, except LUM–H, showed a remarkable in vitro capacity to scavenge DPPH radicals and chelate iron (II) ions. The addition of hydrolysates into 5%wt fish oil-in-water emulsions stabilized with 1%wt Tween20 did not lead to physical destabilization among all the storage time. The latter was observed as emulsions displayed a moderate negative surface charge of the dispersed oil droplets with an average mean diameter D[4,3] of 0.411 ± 0.010 μm at the end of the storage. Regarding their oxidative stability, LUM–H seems to act as a pro-oxidant, promoting emulsion oxidation, which could be attributed to its lowest in vitro antioxidant activity as well as its more negative zeta potential that may favor the attraction of metal ions to the interface, therefore promoting lipid oxidation. In opposition to this result, OSM–H, SFSM–H and RSM–H were able to delay the formation of first and second oxidation products in the emulsions compared with the negative control. Over them, OSM–H and SFSM–H enhanced the oxidative stability of fish oil-in-water emulsions compared with the emulsions with WPC–H and without antioxidants, as shown by AV results. Based on this investigation, enzymatic hydrolysates from vegetable sub-products such as OSM and SFSM could be suitable as bioactive ingredients to incorporate into oil-in-water food emulsions, conferring added value to these substrates from oilseed industries. Additional investigations are needed to study the effect of hydrolysis conditions (e.g., substrate pretreatment, enzymes and degree of hydrolysis) and emulsion preparation (e.g., pH and concentration of hydrolysate) on oxidative stability.

## Figures and Tables

**Table 1:** Percentage solubilization and proximate composition of the plant protein hydrolysates.

Plant Protein Hydrolysate	LUM–H	OSM–H	SFSM–H	RSM–H
Plant meal solubilization (%)	57.9 ± 2.5 ^b^	60.4 ± 1.6 ^b^	67.0 ± 2.8 ^b^	75.5 ± 1.4 ^a^
Protein recovery (%)	56.8 ± 7.0 ^b^	56.6 ± 6.6 ^b^	82.2 ± 11.2 ^a^	79.6 ± 8.9 ^a^
Protein content (%wt)	45.1 ± 3.1 ^a^	25.0 ± 1.6 ^b^	44.7 ± 3.5 ^a^	50.6 ± 3.6 ^a^
Crude fat (%wt)	5.9 ± 0.5 ^b^	8.2 ± 0.8 ^a^	1.7 ± 0.2 ^b^	2.1 ± 0.2 ^b^
Moisture (%wt)	6.9 ± 0.5 ^c^	9.8 ± 0.7 ^a^	9.3 ± 0.6 ^ab^	8.0 ± 0.6 ^bc^

LUM–H: lupin meal hydrolysate; OSM–H: olive seed meal hydrolysate; SFSM–H: sunflower seed meal hydrolysate; RSM–H: rapeseed meal hydrolysate. All the data are expressed as mean ± standard deviation of triplicate measurements. Different superscript letters indicate significant differences (*p* < 0.05) among plant protein hydrolysates.

**Table 2:** Surface net charge (on day 1) and droplet size mean diameters (on day 14) for the emulsions studied.

Emulsion	ζ-Potential (mV)	Surface Mean Diameter D[3,2] (µm)	Volume Mean Diameter D[4,3] (µm)
LUM–H	−48.5 ± 2.70 ^c^	0.316 ± 0.002 ^a^	0.417 ± 0.001 ^ab^
OSM–H	−52.3 ± 2.48 ^c^	0.315 ± 0.005 ^a^	0.393 ± 0.009 ^b^
SFSM–H	−42.7 ± 0.58 ^b^	0.326 ± 0.009 ^a^	0.425 ± 0.021 ^a^
RSM–H	−42.4 ± 0.88 ^b^	0.329 ± 0.009 ^a^	0.418 ± 0.007 ^ab^
WPC–H	−40.1 ± 2.26 ^ab^	0.312 ± 0.007 ^a^	0.399 ± 0.005 ^ab^
NC	−36.1 ± 1.12 ^a^	0.324 ± 0.006 ^a^	0.417 ± 0.005 ^ab^

LUM–H: lupin meal hydrolysate; OSM–H: olive seed meal hydrolysate; SFSM–H: sunflower seed meal hydrolysate; RSM–H: rapeseed meal hydrolysate; WPC–H: whey protein concentrate hydrolysate; NC: negative control (without hydrolysate). All the data are expressed as mean ± standard deviation of triplicate measurements. Different superscript letters indicate significant differences (*p* < 0.05) among plant protein hydrolysates.

## Data Availability

Data is contained within the article and Supplementary Material.

## Supplementary Materials

The following supporting information can be downloaded at: https://www.mdpi.com/article/10.3390/antiox11081612/s1, Figure S1: Image of the fish oil emulsions at day 0; Figure S2: Image of the fish oil emulsions at day 14; Figure S3: Droplet size distribution of the fish oil emulsions at the beginning and end of the storage period.
