# Supplementary figures and images for: Evaluation of Plant Protein Hydrolysates as Natural Antioxidants in Fish Oil-In-Water Emulsions
Source: Antioxidants (Basel). 2022 Aug 19;11(8):1612. doi: 10.3390/antiox11081612 (PMC9404908; doi:10.3390/antiox11081612)

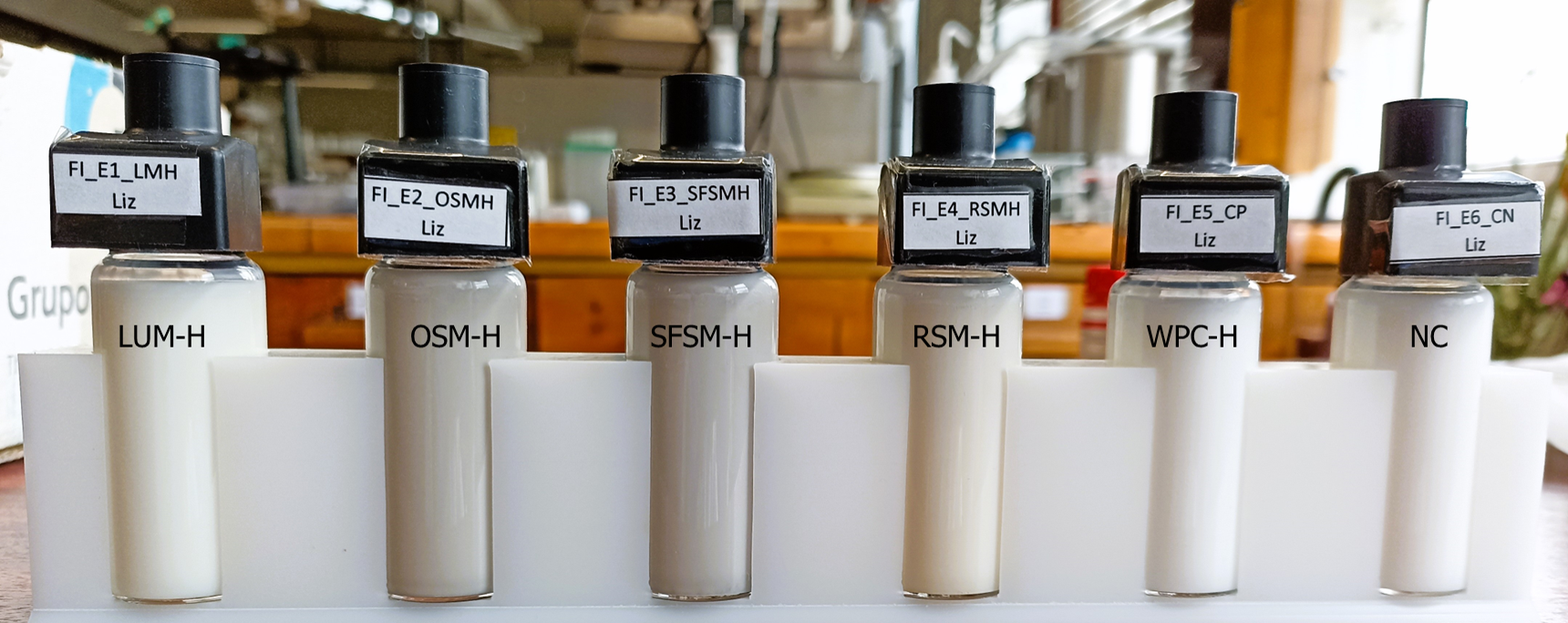

Supplement: Supplementary file 1 [file antioxidants-11-01612-s001.zip › antioxidants-1862405-supplementary/S1.png]

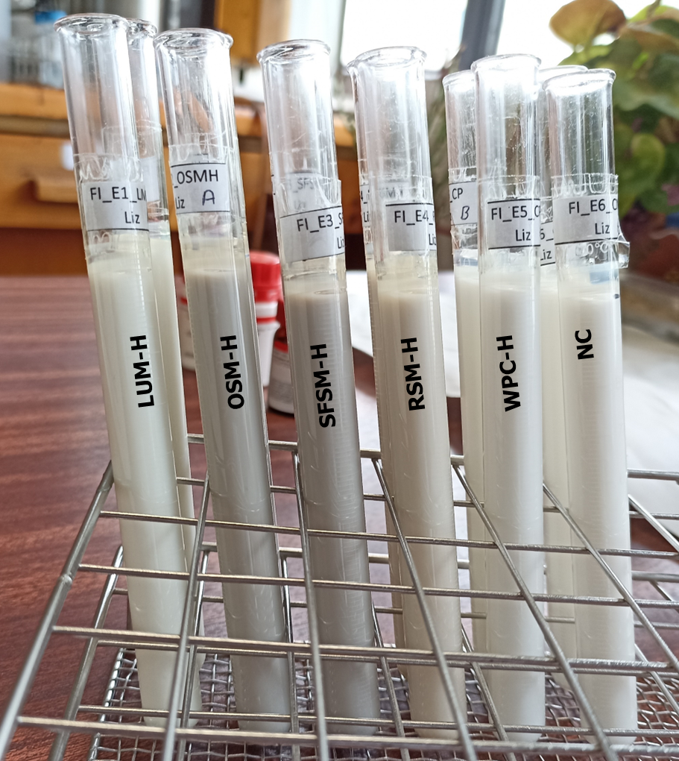

Supplement: Supplementary file 1 [file antioxidants-11-01612-s001.zip › antioxidants-1862405-supplementary/S2.png]

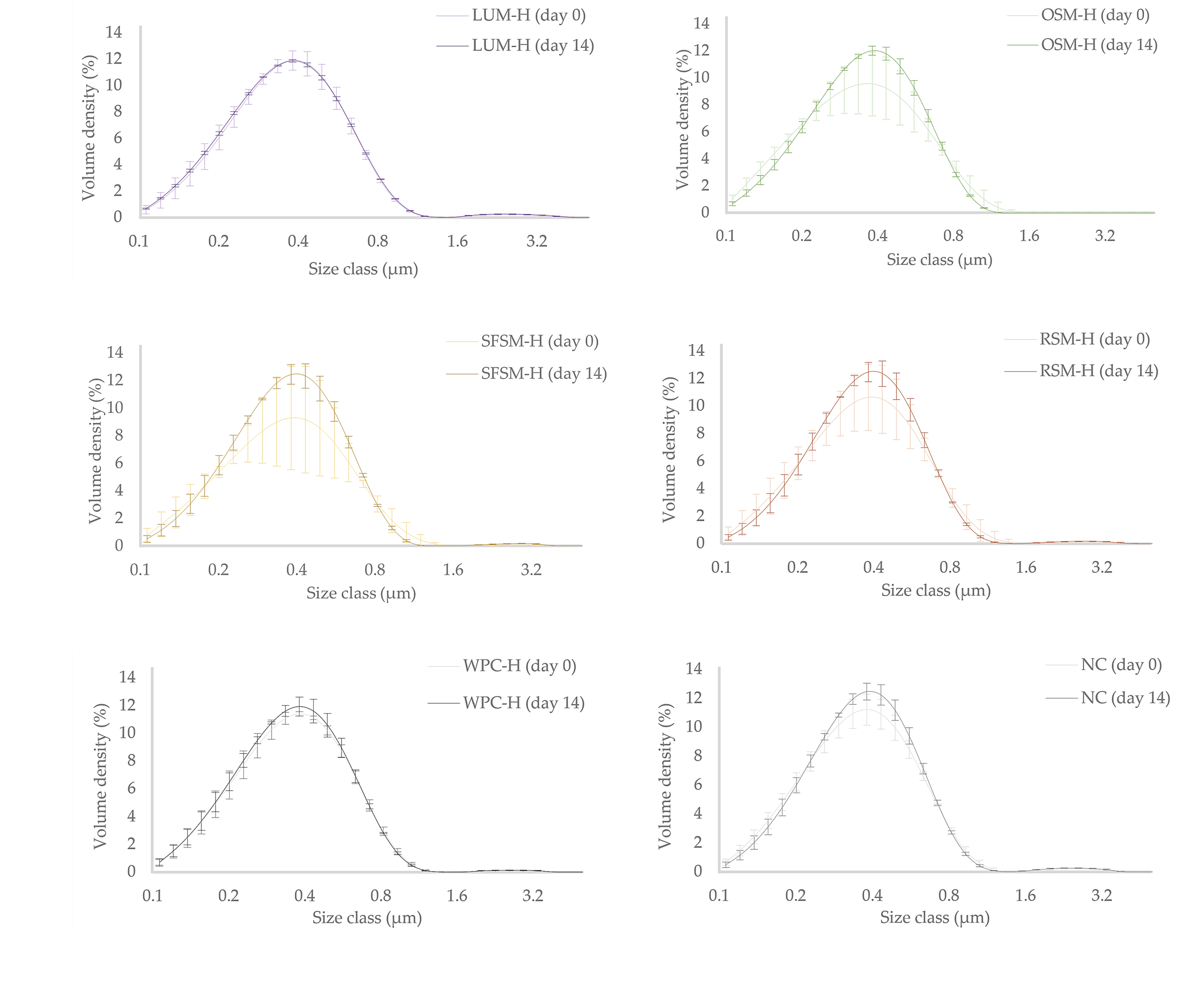

Supplement: Supplementary file 1 [file antioxidants-11-01612-s001.zip › antioxidants-1862405-supplementary/S3.png]
